# Poly I:C enhances cycloheximide-induced apoptosis of tumor cells through TLR3 pathway

**DOI:** 10.1186/1471-2407-8-12

**Published:** 2008-01-17

**Authors:** Qun Jiang, Haiming Wei, Zhigang Tian

**Affiliations:** 1 Institute of Immunology, Hefei National Laboratory for Physical Sciences at Microscale and School of Life Sciences, University of Science and Technology of China, Hefei 230027, P.R. China

## Abstract

**Background:**

Toll-like receptor 3 (TLR3) is a critical component of the innate immune response to dsRNA viruses, which was considered to be mainly expressed in immune cells and some endothelial cells. In this study, we investigated the expression and proapoptotic activity of TLR3 in human and murine tumor cell lines.

**Methods:**

RT-PCR and FACS analysis were used to detect expression of TLR3 in various human and murine tumor cell lines. All tumor cell lines were cultured with poly I:C, CHX, or both for 12 h, 24 h, 72 h, and then the cell viability was analyzed with CellTiter 96^® ^AQueous One Solution, the apoptosis was measured by FACS with Annexin V and PI staining. Production of Type I IFN in poly I:C/CHX mediated apoptosis were detected through western blotting. TLR3 antibodies and IFN-β antibodies were used in Blockade and Neutralization Assay.

**Results:**

We show that TLR3 are widely expressed on human and murine tumor cell lines, and activation of TLR3 signaling in cancerous cells by poly I:C made Hela cells (human cervical cancer) and MCA38 cells (murine colon cancer) become dose-dependently sensitive to protein synthesis inhibitor cycloheximide (CHX)-induced apoptosis. Blockade of TLR3 recognition with anti-TLR3 antibody greatly attenuated the proapoptotic effects of poly I:C on tumor cells cultured with CHX. IFN-β production was induced after poly I:C/CHX treatment and neutralization of IFN-β slightly reduced poly I:C/CHX -induced apoptosis.

**Conclusion:**

Our study demonstrated the proapoptotic activity of TLR3 expressed by various tumor cells, which may open a new range of clinical applications for TLR3 agonists as an adjuvant of certain cancer chemotherapy.

## Background

Toll-like receptor 3 (TLR3) is the critical sensor of the innate immune system that serves to identify viral double-stranded RNA (dsRNA). TLR3 was reported to be expressed on immune cells and some certain noninmmune cells, such as keratinocytes [1] or endothelial cells [2]. TLR3 agonist polyinosinic-polycytidilic acid (poly I:C) represents either genomic or life cycle intermediate material of many viruses, and activates the immune cells through binding both to the dsRNA-dependent protein kinase (PKR) and TLR3. Double-stranded RNA has been proved to induce apoptosis in several cell types through multiple pathways. For instance, dsRNA-transfected pancreatic β-cells manifests PKR- and caspase-dependent apoptosis [3,4], whereas endothelial cell apoptosis triggered by exogenous dsRNA is mostly dependent on the extrinsic caspase pathway [5,6]. As involvement of Toll/IL-1R domain-containing adapter inducing IFN-β (TRIF) in apoptosis has recently been suggested [7,8], TLR3 signaling pathway is found to not only participate in limiting virus replication but also cause infected cells to undergo apoptosis, which is another way of protecting the host against microbe spreading [9].

With the aim of inducing an IFN-mediated anticancer immune response, both poly I:C and poly A:U have been used with moderate success as adjuvant therapy in clinical trials for different types of cancer [10,11]. Recently, Bruno and his colleagues reported that TLR3 was expressed in several breast cancer cell lines and could directly drive those cells to apoptosis [12]. Here, we extensively analyzed the expression and proapoptotic activity of TLR3 in a variety of human and murine tumor cells, and further confirmed that TLR3 are widely expressed on human and murine tumor cells. We then found that activation of TLR3 signaling in cancerous cells by poly I:C made human and murine cancer cells become sensitive to protein synthesis inhibitor cycloheximide (CHX)-induced apoptosis, and blockade of TLR3 recognition with anti-TLR3 antibody greatly attenuated the apoptosis-improving effects of poly I:C on tumor cells.

## Methods

### Cell Lines and Reagents

The human tumor cell lines Hela (cervical cancer), A549 (small cell lung carcinoma), Hep2 (laryngeal carcinoma), HepG2 (hepatoma), HO8910 (ovarian epithelial carcinoma), and the murine cell lines B16 (melanoma), RM1 (prostate cancer), LLC (lung cancer), MCA38 (colon cancer), Hepa1-6 (hepatocellular carcinoma) were obtained from American Type Culture Collection (ATCC, Rockville, MD, U.S.A.). Polyinosinic-polycytidilic acid (poly I:C) and cycloheximide (CHX) was purchased from Sigma-Aldrich Co. Ltd (St. Louis, MO, USA).

### Cell Cultures

Tumor cells were maintained in 2 ml RPMI 1640 plus 10% (v/v) heat-inactivated fetal bovine serum (FCS, GIBCO, Grand Island, NY) in 6-well plates (Costar, Austria) at 2 × 10^5 ^cells/well. All of the media were supplemented with 2 mML-glutamine, 100 units/ml penicillin G, 100 units/ml streptomycin. Cells were maintained at 37°C in a humidified incubator containing 5% CO_2_. Poly I:C was used at the concentrations indicated. CHX was added to the media at the concentration of 1.5 μg/ml.

### RT-PCR Analysis

Total RNA was isolated from tumor cells using TRIzol reagent according to manufacture's guide (Invitrogen, Carlsbad, CA). Cellular RNA (1 μg) was reversedly transcribed into cDNA in a reaction mixture containing 5 mM MgCl2, 1 mM dNTP, 2.5 μM oligo (dT) primer, 1U RNase inhibitor, and 2.5U reverse transcriptase (Invitrogen). After incubation at 37°C for 50 min, the reaction was terminated by heating at 70°C for 15 min. PCR primers for detecting mRNA for TLR3 and β-actin were synthesized by Sangon Ltd, Shanghai, China. Primer sequences were as follows: human β-actin, sense, 5'-GTG GGG CGC CCC AGG CAC CA-3', antisense 5'-CTC CTT AAT GTC ACG CAC GAT TT-3'; human TLR3, sense, 5'-AAC GAT TCC TTT GCT TGG CTT C-3', antisense 5'-GCT TAG ATC CAG AAT GGT CAA G-3'; mouse β-actin, sense, 5'- ATG GAT GAC GAT ATC GCT -3', antisense, 5'- ATG AGG TAG TCT GTC AGG T -3'; mouse TLR3, sense, 5'-AAG AGG GCG GAA AGG TG-3', antisense, 5'-GAA GCG AGC ATT TAC TA-3'. The PCR reaction buffer (25 μl), consisting of 2 mM MgCl_2_, 0.5 μM of each primer, and 1U Ampli Taq DNA polymerase, was added to an amplification tube. PCR was run for 35 cycles. Each cycle consisted of 95°C for l min, 55°C for l min, and 72°C for l min.

### Flow Cytometric Analysis

To detect cell surface expression, cultured human tumor cells were stained with purified anti-human TLR3 antibody (eBioscience, San Diego, CA) or purified Mouse IgG1 control (eBioscience), followed by FITC-conjugated rat anti-mouse IgG1 mAb (clone A85-1, BD PharMingen, San Diego, CA). The Murine tumor cells were stained with rat serum anti-mouse TLR3 (eBioscience) or purified rat IgG isotype control (eBioscience), followed by FITC-conjugated F(ab')2 goat anti-rat IgG (Caltag Laboratories, South San Francisco, CA). To analyze intracytoplasmic TLR3 expression, tumor cells were prefixed and permeabilized. The following staining treatments were the same as above. Finally, stained cells were analyzed by using a flow cytometer (FACScalibur, Becton Dickinson, Franklin Lakes, NJ, USA), and the data were processed with WINMDI2.9 software.

### Cell Viability Analysis

Cell viability was determined by Cell-titer 96 aqueous one solution cell proliferation assay kit (Promega, Madison, WI, USA). The process of the experiment is completely according to the instruction. Briefly, aliquots of 1 × 10^3 ^cells/well were cultured in 96-well plates (Costar, Austria) with or without Poly I:C/CHX for 72 h. Poly I:C was used at the concentrations of 100 μg/ml. CHX was added at the concentrations of 1.5 μg/ml. 40 μl of Cell-titer 96 aqueous one solution were added to each well and incubated for an additional 3 h. The absorbance at 490 nm was recorded with a 96-well plate reader. Each experiment was performed in triplicate and repeated at least three times.

### Apoptosis Assays

2 × 10^5 ^tumor cells were washed twice with cold PBS, followed by being resuspended in 100 binding buffer (10 mM HEPES-NaOH, pH 7.4, 140 mM NaCl, 2.5 mM CaCl_2_) and then incubated with 4 μl FITC-conjugated annexin-V and 5 μl Propidium iodide (PI) from BD PharMingen (San Diego, CA) in dark for 20 min at room temperature. Samples were immediately analyzed with flow cytometry. The stained cells were also analyzed by a FACScalibur flow cytometer (Becton Dickinson).

### Western Blot Analysis

Hela cells and MCA38 cells were stimulated with poly I:C/CHX for 3 hours, 6 hours, 9 hours or 12 hours respectively. Cellular extracts were prepared as described [13]. Protein samples were mixed in Laemmli loading buffer, boiled for 5 min, and then subjected to 14% SDS-PAGE. After electrophoresis, proteins were transferred onto PVDF membrane (Millipore, Billerica, MA). The blots were incubated with rabbit anti-mouse or human IFN-β polyclonal antibody (PBL Biomedical Laboratories) overnight at 4°C. Membranes were washed with 0.05% (vol/vol) Tween 20 in PBS (pH 7.6) and incubated with a 1:3000 dilution of Horseradish peroxidase (HRP) linked anti-rabbit IgG secondary antibody (Promega) for 60 min at room temperature. Protein bands were visualized by ECL substrate (Pierce).

### TLR3 Blockade and IFN-β Neutralization Assay

Hela cells and MCA38 cells were maintained in 12-well plates (Costar, Austria) at 1 × 10^5 ^cells/well. In TLR3 Blockade assay, Hela cells were treated with the purified anti-human TLR3 antibody (eBioscience) at the concentration of 10 μg/ml for 4 hours before being stimulated with poly I:C/CHX. The treatment of MCA38 cells is identical to Hela cells except for the rat serum anti-mouse TLR3 antibody (eBioscience) we used. In IFN-β Neutralization assay, Hela cells or MCA38 cells were treated with anti-human IFN-β antibody (PBL Biomedical Laboratories) or anti-mouse IFN-β antibody (PBL Biomedical Laboratories) at the concentration of 1 × 10^4 ^U/ml for 4 hours before being stimulated with poly I:C/CHX.

### Statistical Analysis

Data are expressed as the means ± SD with n = 3. Statistical significances were determined with use of the unpaired Student's *t *test (P values < 0.05). All data from cell culture experiments are on the basis of at least three individual cell preparations.

## Results

### Toll-like receptor3 was widely expressed in human and murine tumor cells

**Figure 1:**
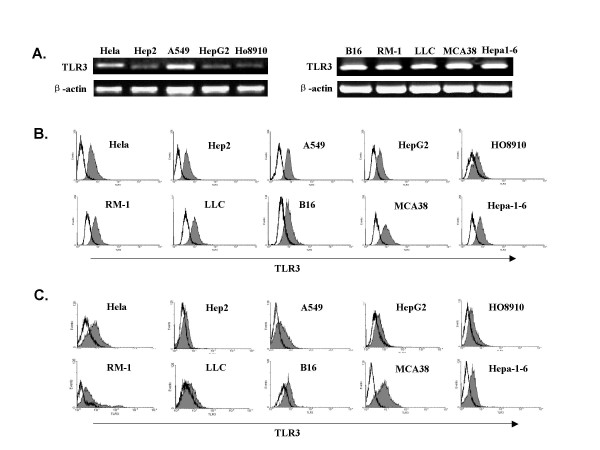
**Expression of TLR3 in tumor cells**. (A). Expression of TLR3 in different human and mouse tumor cell lines was analyzed by RT-PCR. One representative experiment (out of three) is depicted. (B, C). FACS analysis of intracellular(B) and cell surface(C) expression of TLR3 in tumor cell lines. Histograms showed TLR3 expression (shadow area) with isotype controls (black lines).

we screened various human and murine tumor cell lines from different tissues, including human cell line Hela (cervical cancer), A549 (lung carcinoma), Hep2 (laryngeal carcinoma), HepG2 (hepatoma), HO8910 (ovarian epithelial carcinoma), and murine cell line B16 (melanoma), RM1(prostate cancer), LLC (lung cancer), MCA38 (colon cancer), Hepa1-6 (hepatocellular carcinoma) for the expression of TLR3 by RT-PCR (Fig 1A) and FACS analysis (Fig 1B, C). We found that all these tumor cell lines expressed TLR3 mRNA. FACS analysis showed that TLR3 was present both inside the cells and on the cell surface, but the expression on the protein level is distinctly higher in Hela, A549 and MCA38 cells.

### Poly I:C treatment caused tumor cells more sensitive to CHX-induced cell death

**Figure 2:**
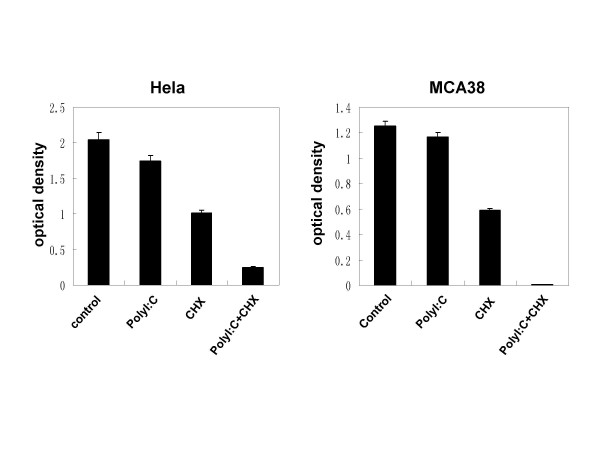
**Tumor cell viability after treatment with poly I:C and CHX**. Tumor cells were cultured with poly I:C (100 μg/ml) alone, CHX (1.5 μg/ml) alone or poly I:C plus CHX for 72 h. 1×PBS was added as control. 40 μl of CellTiter 96^® ^AQueous One Solution were added to each well and incubated for additional 3 hrs before final examination with absorbance at 490 nm. Optical density value shows the viability of cells tested. Results are from one representative experiment of three repeated experiments.

To investigate the effects of TLR3 on tumor cells, all tumor cell lines were pre-cultured with 100 μg/ml of poly I:C, a ligand of TLR3, for 72 h, and then cell viability was analyzed. Unexpectedly, few tumor cell lines tested showed a significant decrease in cell viability after treatment with poly I:C alone (all less than 10%, data not shown). However, cell viability of protein synthesis inhibitor cycloheximide (CHX)-treated tumor cells (Hela cells and MCA38 cells) became dramatically lower in the presence of poly I:C (Fig 2). We also compared the expression level of TLR3 in poly I:C- or CHX- treated Hela cells and MCA38 cells with PBS-treated control cells, but found no remarkable changes (see Additional file 1).

### Poly I:C dose-dependently increased CHX-induced apoptosis of tumor cells

**Figure 3:**
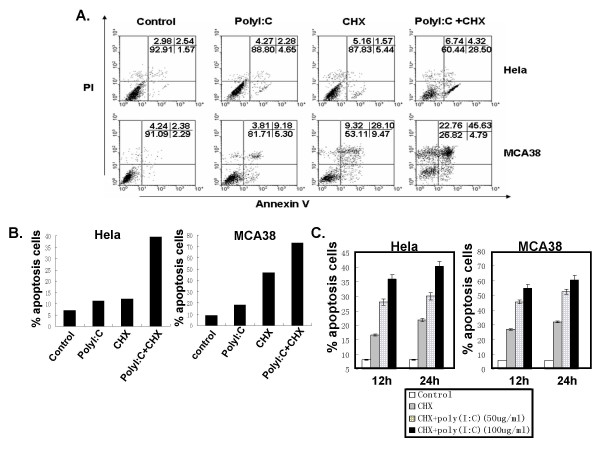
**Tumor cell apoptosis after treatment with poly I:C and CHX**. (A) FACS analysis of apoptosis in Hela and MCA38 cells after stimulation with poly I:C and CHX. Tumor cells were cultured for 24 h with PBS, poly I:C (100 μg/ml), CHX (1.5 μg/ml) or poly I:C puls CHX, and apoptosis was measured by Annexin V, PI staining. (B) Statistic analysis of apoptotic cells based on Fig 1A. (C) The dynamic analysis of apoptosis of tumor cells. Cancer cells (Hela or MCA38) were cultured with different concentrations of poly I:C (50 or 100 μg/ml) and the same concentration of CHX (1.5 μg/ml) for 12 h or 24 h. PBS was used as control. Apoptosis was measured by flow cytometry with anti-Annexin V and anti-PI antibodies. Data were obtained from three independent experiments.

We then examined the apoptosis of a variety of human and murine tumor cells by double-staining of Annexin V and PI. All tumor cells incubated in poly I:C alone even in high concentrations (up to 100 μg/ml) showed minimal apoptosis. Likewise, CHX alone (1.5 μg/ml) had no significant effect on these tumor cell lines. However, when incubated in the presence of both CHX and Poly I:C, the Annexin V/PI positive tumor cells significantly increased in the Hela cells and MCA38 cells (Hela and MCA38 up to about 40% and 70% respectively, Fig 3A, 3B), and few other tumor cell lines tested showed notable differences between apoptosis of tumor cells stimulated with CHX alone or poly I:C/CHX (data not shown). We still found that the induction of the apoptosis was dose-dependent but not time-dependent (Fig 3C). These findings suggested that tumor cells death is related to poly I:C-improved CHX-induced apoptosis.

### Blockade of TLR3 recognition attenuated the apoptosis-improving effects of poly I:C on tumor cells

**Figure 4:**
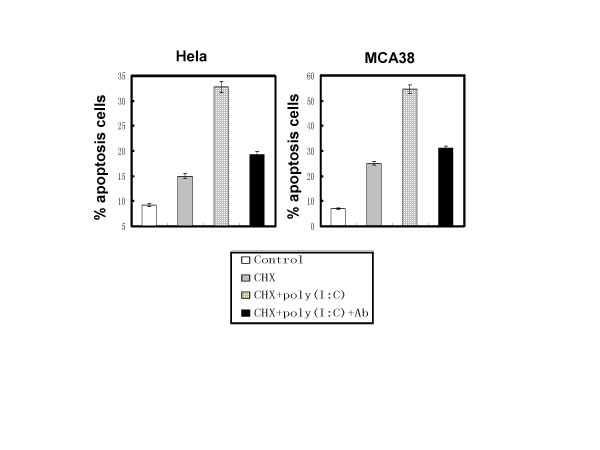
**TLR3 blockade attenuates poly I:C-improved tumor apoptosis**. Hela cells or MCA38 cells were treated with purified anti-human TLR3 antibody or anti-mouse TLR3 antibody at the concentration of 10 μg/ml for 4 hours before being stimulated with poly I:C (100 μg/ml)/CHX (1.5 μg/ml) for 12 hours. Apoptosis were determined as above. The data is presented as the mean ± SD.

As double-stranded RNA has also been proved to induce cell apoptosis through PKR pathway, we use antibody to block TLR3 signaling in order to prove that the poly I:C-enhanced tumor cells apoptosis is mediated by TLR3. The results showed that blockade of TLR3 recognition with anti-TLR3 antibody greatly attenuated the apoptosis-improving effects of poly I:C on tumor cells (Fig 4).

### Type I IFN was involved in the apoptosis-improving effects of poly I:C on tumor cells

**Figure 5:**
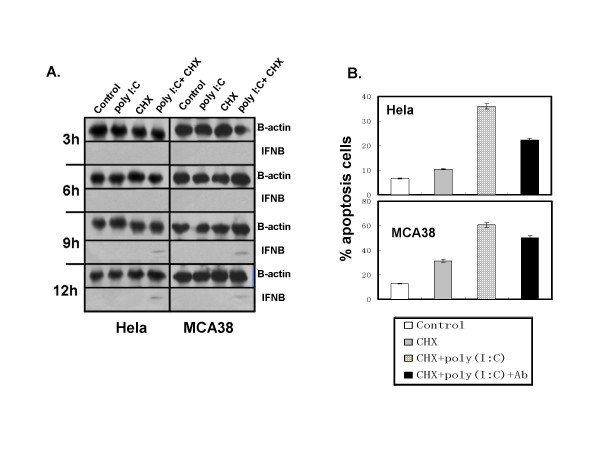
**Neutralization of IFN-β partly attenuates poly I:C-improved tumor apoptosis**. (A) IFN-β protein level is measured by WB in lysates of Hela cells or MCA38 cells cultured with poly I:C/CHX for the indicated time periods. β-actin is shown as a loading control. (B) Hela cells or MCA38 cells were treated with anti-human IFN-β antibody or anti-mouse IFN-β antibody at the concentration of 1 × 10^4 ^U/ml for 4 hours before being stimulated with poly I:C (100 μg/ml)/CHX (1.5 μg/ml) for 12 hours. Apoptosis were determined as above. The data is presented as the mean ± SD.

Because several studies reported that the production of IFN-β was required in cell apoptosis induced by TLR3 agonists [14,15], we evaluated the role of type I IFN in poly I:C/CHX mediated apoptosis. Western blot result showed that both human and murine IFN-β productions of Hela cells and MCA38 cells were mildly induced after 9 h of poly I:C/CHX treatment (Fig 5A). Neutralization of IFN-β with antibodies since the tumors were stimulated slightly reduced poly I:C/CHX -induced apoptosis (Fig 5B), suggesting that IFN-β was involved in TLR3-triggered cytotoxicity.

## Discussion

TLR3 was thought to be mainly expressed in immune cells, keratinocytes and some endothelial cells. Recently, it has been reported that certain human tumor cells also express this receptor [12,16]. Our work shows that TLR3 is widely expressed in human and murine tumor cells lines from different origin though at different levels, suggesting that TLR3 activation may play important functions in tumor biology.

Ligation of TLR3 and its ligand dsRNA triggers well-characterized signaling cascades that result in activation of downstream effectors, such as NF-κB, p38, JNK, and IFN regulatory factors (IRFs) 4 [17]. Many of these signaling elements are also involved in tumor growth and apoptosis, implying that TLR3 expressed in tumor cells may also affect tumor viability. In the present study, we investigated the response of tumor cell lines from different origin in vitro to the stimulation with TLR3 agonist poly I:C and the tumor chemotherapeutic drug CHX. We found that incubating tumor cells with CHX alone in the used dosage or poly I:C alone showed no significant effects on cancer cell viability. However, incubation of human Hela cells or murine MCA38 cells with CHX plus poly I:C caused dramatically apoptosis of these cells in a poly I:C dose-dependent manner via TLR3 pathway, which is confirmed by our TLR3 blockade assay. Several studies have reported that molecular events involved in cell death induced by TLR3 agonists include the production of IFN-β[14,15] required for apoptosis. In our study, we also found that IFN-β production of tumor cells could be induced by poly I:C/CHX stimulation, which might function as a proapoptotic agent by up-regulating the expression of proteins directly involved in cell death, including caspases [18], TRAIL [19,20], and p53 [21] as reported. On the other hand, partly reduction of poly I:C/CHX -induced apoptosis in the neutralization assay of IFN-β suggested other pathway involved. Thus roles of proteins such as TBK1 and RIP1, which all participate in TLR3 signaling [22] will require additional studies. Since Salaun has recently proved that in contrasts with its survival role after TLR2 [23]and TLR4 [24] triggering, NF-κB appears to be necessary for TLR3-mediated apoptosis [12], it would be interesting to investigate how NF-κB links TLR3 triggering and CHX stimulating to apoptosis pathway.

Besides TLR3, PKR, the cytoplasmic helicase family proteins (retinoic acid-inducible gene I (RIG-I) and melanoma differentiation-associated gene 5 (MDA5)) also serve as dsRNA pattern-recognition receptors [1,25], which are reported to trigger different signaling pathways from TLR3 [26]. Although blockade of TLR3 markedly reduced apoptosis of tumor cells treated by poly I:C/CHX, we can not completely exclude the possibility that RIG-I/MDA5 contributed to the recognition of poly I:C internalized by endocytosis. Moreover, unpublished result of our study on the apoptosis in other tumor cell lines transfected with vector expressing poly I:C demonstrated the involvement of RIG-I/MDA5 pathway.

It is still largely unknown how CHX induces tumor cells cytotoxicity in the presence of poly I:C, although we figured out the production of IFN-β involved. Furthermore, there were still some cell lines we tested insensitive to poly I:C, CHX or both, which can be explained by defects in the cellular apoptotic machinery or low expression levels of TLR3.

Since both poly I:C and poly(A:U) have been used with moderate success as immune adjuvant therapy in clinical trials for different types of cancer [27], including adenocarcinomas of the breast [28], our findings may open a new range of the applications for TLR3 agonists as an adjuvant of cancer chemotherapy.

## Conclusion

We have described the proapoptotic activity of TLR3 expressed by various tumor cells, uncovered the association of TLR3 signaling with protein synthesis inhibition in tumor cells. Our study may open a new range of therapeutic applications for TLR3 agonists as a adjuvant of chemotherapy drugs in some certain cancers.

## Competing interests

The author(s) declare that they have no competing interests.

## Authors' contributions

QJ carried out the experiments and drafted the primary manuscript. HW and ZT addressed the scientific question, designed the experiments, analyzed the data and finally wrote the manuscript. All authors read and approved the final manuscript.

## Pre-publication history

The pre-publication history for this paper can be accessed here:



## Supplementary Material

Additional file 1 Supplementary figure 1: Analysis of poly I:C and CHX effects on TLR3 expression in Hela cells and MCA38 cells. (A) mRNA levels of TLR3 in Hela cells and MCA38 cells treated with poly I:C (100 μg/ml) for 72 hours were detected by RT-PCR. (B) FACS analysis of intracellular TLR3 in Hela cells and MCA38 cells treated with CHX (2.5 μg/ml) for 24 hours was shown. Click here for file

## References

[B1] Yoneyama M, Kikuchi M, Natsukawa T, Shinobu N, Imaizumi T, Miyagishi M, Taira K, Akira S, Fujita T (2004). The RNA helicase RIG-I has an essential function in double-stranded RNA-induced innate antiviral responses. Nat Immunol.

[B2] Miettinen M, Sareneva T, Julkunen I, Matikainen S (2001). IFNs activate toll-like receptor gene expression in viral infections. Genes Immun.

[B3] Scarim AL, Arnush M, Blair LA, Concepcion J, Heitmeier MR, Scheuner D, Kaufman RJ, Ryerse J, Buller RM, Corbett JA (2001). Mechanisms of beta-cell death in response to double-stranded (ds) RNA and interferon-gamma: dsRNA-dependent protein kinase apoptosis and nitric oxide-dependent necrosis. Am J Pathol.

[B4] Robbins MA, Maksumova L, Pocock E, Chantler JK (2003). Nuclear factor-kappaB translocation mediates double-stranded ribonucleic acid-induced NIT-1 beta-cell apoptosis and up-regulates caspase-12 and tumor necrosis factor receptor-associated ligand (TRAIL). Endocrinology.

[B5] Kaiser WJ, Kaufman JL, Offermann MK (2004). IFN-alpha sensitizes human umbilical vein endothelial cells to apoptosis induced by double-stranded RNA. J Immunol.

[B6] Sato A, Iizuka M, Nakagomi O, Suzuki M, Horie Y, Konno S, Hirasawa F, Sasaki K, Shindo K, Watanabe S (2006). Rotavirus double-stranded RNA induces apoptosis and diminishes wound repair in rat intestinal epithelial cells. J Gastroenterol Hepatol.

[B7] Ruckdeschel K, Pfaffinger G, Haase R, Sing A, Weighardt H, Hacker G, Holzmann B, Heesemann J (2004). Signaling of apoptosis through TLRs critically involves toll/IL-1 receptor domain-containing adapter inducing IFN-beta, but not MyD88, in bacteria-infected murine macrophages. J Immunol.

[B8] Kaiser WJ, Offermann MK (2005). Apoptosis induced by the toll-like receptor adaptor TRIF is dependent on its receptor interacting protein homotypic interaction motif. J Immunol.

[B9] Everett H, McFadden G (1999). Apoptosis: an innate immune response to virus infection. Trends Microbiol.

[B10] Lacour J, Lacour F, Spira A, Michelson M, Petit JY, Delage G, Sarrazin D, Contesso G, Viguier J (1980). Adjuvant treatment with polyadenylic-polyuridylic acid (Polya.Polyu) in operable breast cancer. Lancet.

[B11] Khan AL, Heys SD, Eremin O (1995). Synthetic polyribonucleotides: current role and potential use in oncological practice. Eur J Surg Oncol.

[B12] Salaun B, Coste I, Rissoan MC, Lebecque SJ, Renno T (2006). TLR3 can directly trigger apoptosis in human cancer cells. J Immunol.

[B13] Ihara S, Nakajima K, Fukada T, Hibi M, Nagata S, Hirano T, Fukui Y (1997). Dual control of neurite outgrowth by STAT3 and MAP kinase in PC12 cells stimulated with interleukin-6. Embo J.

[B14] Tanaka N, Sato M, Lamphier MS, Nozawa H, Oda E, Noguchi S, Schreiber RD, Tsujimoto Y, Taniguchi T (1998). Type I interferons are essential mediators of apoptotic death in virally infected cells. Genes Cells.

[B15] Chawla-Sarkar M, Lindner DJ, Liu YF, Williams BR, Sen GC, Silverman RH, Borden EC (2003). Apoptosis and interferons: role of interferon-stimulated genes as mediators of apoptosis. Apoptosis.

[B16] Khvalevsky E, Rivkin L, Rachmilewitz J, Galun E, Giladi H (2007). TLR3 signaling in a hepatoma cell line is skewed towards apoptosis. J Cell Biochem.

[B17] Akira S, Takeda K (2004). Toll-like receptor signalling. Nat Rev Immunol.

[B18] Juang SH, Wei SJ, Hung YM, Hsu CY, Yang DM, Liu KJ, Chen WS, Yang WK (2004). IFN-beta induces caspase-mediated apoptosis by disrupting mitochondria in human advanced stage colon cancer cell lines. J Interferon Cytokine Res.

[B19] Chawla-Sarkar M, Leaman DW, Jacobs BS, Borden EC (2002). IFN-beta pretreatment sensitizes human melanoma cells to TRAIL/Apo2 ligand-induced apoptosis. J Immunol.

[B20] Morrison BH, Tang Z, Jacobs BS, Bauer JA, Lindner DJ (2005). Apo2L/TRAIL induction and nuclear translocation of inositol hexakisphosphate kinase 2 during IFN-beta-induced apoptosis in ovarian carcinoma. Biochem J.

[B21] Takaoka A, Hayakawa S, Yanai H, Stoiber D, Negishi H, Kikuchi H, Sasaki S, Imai K, Shibue T, Honda K, Taniguchi T (2003). Integration of interferon-alpha/beta signalling to p53 responses in tumour suppression and antiviral defence. Nature.

[B22] Barton GM, Medzhitov R (2004). Toll signaling: RIPping off the TNF pathway. Nat Immunol.

[B23] Aliprantis AO, Yang RB, Mark MR, Suggett S, Devaux B, Radolf JD, Klimpel GR, Godowski P, Zychlinsky A (1999). Cell activation and apoptosis by bacterial lipoproteins through toll-like receptor-2. Science.

[B24] Hsu LC, Park JM, Zhang K, Luo JL, Maeda S, Kaufman RJ, Eckmann L, Guiney DG, Karin M (2004). The protein kinase PKR is required for macrophage apoptosis after activation of Toll-like receptor 4. Nature.

[B25] Kato H, Takeuchi O, Sato S, Yoneyama M, Yamamoto M, Matsui K, Uematsu S, Jung A, Kawai T, Ishii KJ, Yamaguchi O, Otsu K, Tsujimura T, Koh CS, Reis e Sousa C, Matsuura Y, Fujita T, Akira S (2006). Differential roles of MDA5 and RIG-I helicases in the recognition of RNA viruses. Nature.

[B26] Li K, Chen Z, Kato N, Gale M, Lemon SM (2005). Distinct poly(I-C) and virus-activated signaling pathways leading to interferon-beta production in hepatocytes. J Biol Chem.

[B27] Seya T, Akazawa T, Uehori J, Matsumoto M, Azuma I, Toyoshima K (2003). Role of toll-like receptors and their adaptors in adjuvant immunotherapy for cancer. Anticancer Res.

[B28] Laplanche A, Alzieu L, Delozier T, Berlie J, Veyret C, Fargeot P, Luboinski M, Lacour J (2000). Polyadenylic-polyuridylic acid plus locoregional radiotherapy versus chemotherapy with CMF in operable breast cancer: a 14 year follow-up analysis of a randomized trial of the Federation Nationale des Centres de Lutte contre le Cancer (FNCLCC). Breast Cancer Res Treat.

