# Supplementary figures and images for: Poly I:C enhances cycloheximide-induced apoptosis of tumor cells through TLR3 pathway
Source: BMC Cancer. 2008 Jan 17;8:12. doi: 10.1186/1471-2407-8-12 (PMC2242792; doi:10.1186/1471-2407-8-12)

## Slide 1
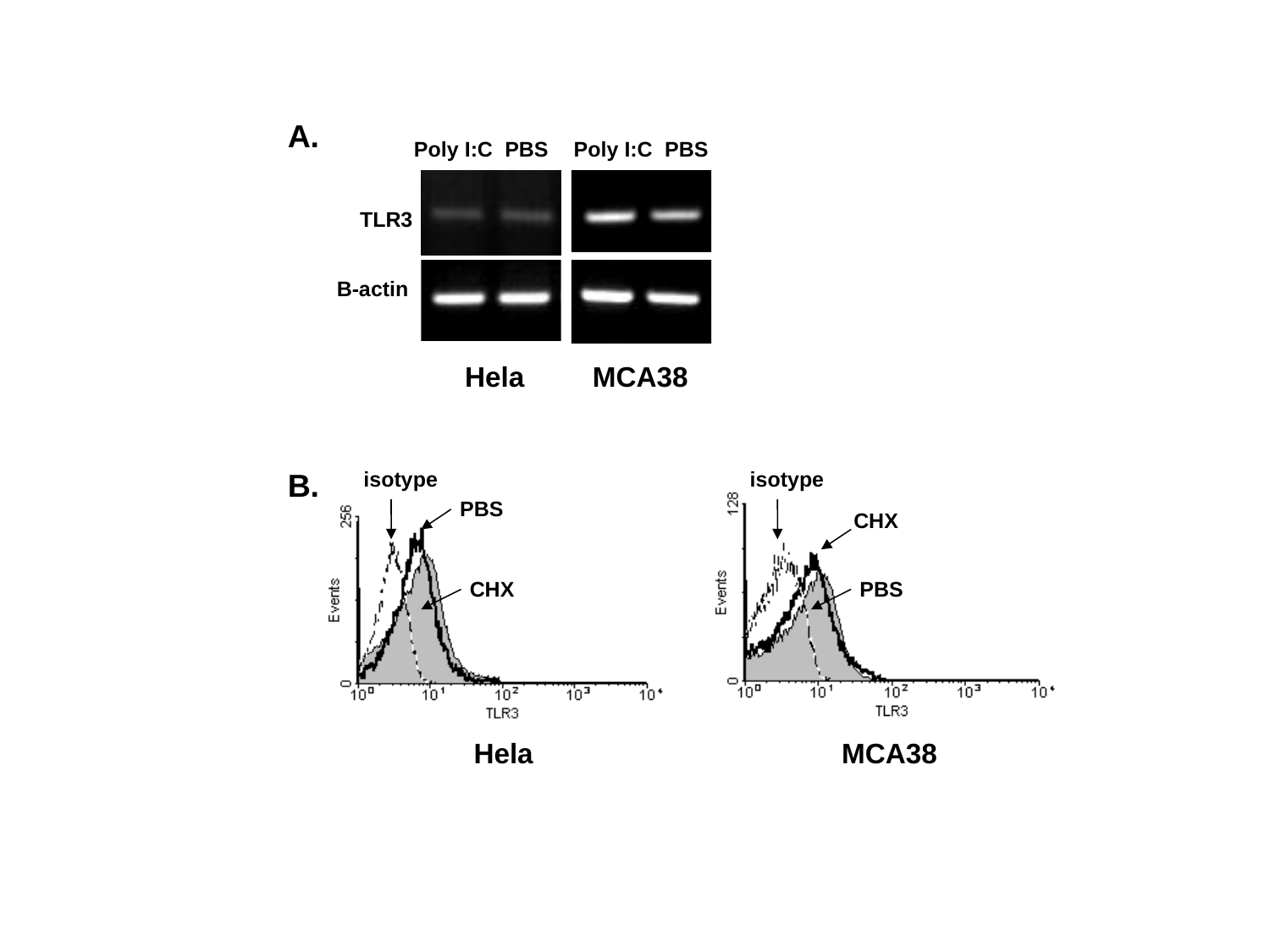

A.
Poly I:C
 PBS
Poly I:C
 PBS
 TLR3
Β-actin
Hela
 MCA38
isotype
isotype
 PBS
 CHX
 CHX
 PBS
Hela
 MCA38
B.

Supplement: Additional file 1 — Supplementary figure 1: Analysis of poly I:C and CHX effects on TLR3 expression in Hela cells and MCA38 cells. (A) mRNA levels of TLR3 in Hela cells and MCA38 cells treated with poly I:C (100 μg/ml) for 72 hours were detected by RT-PCR. (B) FACS analysis of intracellular TLR3 in Hela cells and MCA38 cells treated with CHX (2.5 μg/ml) for 24 hours was shown. [file 1471-2407-8-12-S1.PPT]
